# Set Your Shoulder at the Wheel, to Advance the Business

**Published:** 1915-12-18

**Authors:** 


### The Hospital

The Workers' Newspaper of Administrative Medicine and Institutional Life, Administration, National Insurance and Health.

No. 1540, Vol. LIX. SATURDAY, DECEMBER 18, 1915. PRICE ONE PENNY.

### SET YOUR SHOULDER AT THE WHEEL, TO ADVANCE THE BUSINESS.

No man or woman living in these islands before the present war began had probably any true realisation of what a great war means for all of us. Some of us who are too old to fight have already suffered vicariously by the loss of dear ones whose place can never be filled; others have already nobly given their lives at the call of Empire and country in defence of the rights of the smaller nations and the defenceless ones who have been murdered at the cruel hands of a barbarous foe. They were champions of the principles taught the world by Christ, without which life on this planet must degenerate into a curse for all, through the tyrant and brutalised rulers of the earth. All have felt horror at the misery and unrighteousness resulting from the upholding and enforcement by our enemies of pagan principles, and even worse. So it has come to pass that Christmas Day has been dreaded this year by many; but such dread can have no influence or weight in man or woman who believes and knows that all things happen for good to those who love God.

We would remind the impatient that no one till his death should be called unhappy; it is a feckless and faithless act to measure the work until the day is out and the labour done. So faith will carry us all through existing and future troubles and horrors, for the end is not yet, and by patient endurance in well-doing, standing shoulder to shoulder, we men of England and the Empire, with our women, need have no fear for the future, for it is in the hands of God.

The great thing with every one of us during war-time is to find each his own work; for, as Carlyle declares, " genuine work alone, what thou workest faithfully, that is eternal, as the Almighty Founder and Well Builder himself." Sharing these views, and knowing their absolute soundness from a long life's experience, we have determined to try to give a helping hand to all who are in any way associated, either as workers or as supporters, with our hospitals and charities maintained by voluntary contributions. Our plan is to devote this last Special Number of The Hospital for 1915 to an effort to show many directions in which a multitude of men and women may find the true work for themselves which has something of divineness in it. All such people must realise that the honoured dead, our forbears, leaders, and inspirers, still have left behind them good undone for us who live to do. The fields of work which we present to our readers in this Special Number yield, in many directions, some aim for the heart and the will and the soul of a man or a woman to pursue.

Readers of The Hospital were amongst the first of the workers of the nation to embrace and practise personal service (see p. 261). It is a glorious fact that The Hospital originated, and steadily supported to success, the founding of the Royal National Pension Fund for Nurses, with the object of freeing the trained nurse from any fear of destitution and loss of independence when she grew old, by securing for her a provision on which she could live till her life's end. This Fund has become the greatest thrift organisation for women workers which the world has ever seen. After twenty-eight years it is announced that its invested funds to-day exceed two millions sterling, being nurses' savings, the larger portion of which has been invested in War Loan stock.

We have reason, therefore, to thank God and take courage, and to invite our readers to keep this Special Number, to study it, and through it, with or without our assistance, to find their work and to do it. Then for them, as for all workers, the war will be deprived of most of its terrors, and the future, through faith, will bring to us the assured hope that all will yet be well.

